# A study on the normative path of ethics review in China: based on the perspective of Panopticism

**DOI:** 10.3389/fmed.2023.1268046

**Published:** 2023-10-27

**Authors:** Leqian Wu, Xiangjin Kong

**Affiliations:** College of Humanities and Social Sciences, Dalian Medical University, Dalian, China

**Keywords:** ethical review, Panopticism, sign systems, discipline, punish

## Abstract

Modern biomedical technology is in an era of dramatic development, which brings unprecedented challenges to the work of ethics review and provides a turning point for the construction of ethics review system. The current ethics review committee (ERC) in China is executed with low efficiency and quality, which can hardly meet the current needs of biomedical research involving human beings. This paper summarizes the main connotations and roles of the sign system technique and the discipline mechanism through the idea of Foucault’s Panopticism, and proposes to incorporate the Panopticism into the construction of the ethics review system and establish the sign system and discipline mechanism of ethics review, in order to build an ethics review system and the operation system of the ethics review committee suitable for China’s national conditions.

## 1. Introduction

The “2018 He Jiankui human genome editing case” exposed loopholes in China’s ethics review system, highlighting the need for improvement. As biomedical research involving human subjects becomes more prevalent, the ethical review process has become cumbersome and complex. To regulate life science and medical research involving human subjects and enhance the quality and efficiency of ethical review, the National Health Commission of the People’s Republic of China issued the Measures for Ethical Review of Life Sciences and Medical Research Involving Human Beings (referred to as the Review Measures) on February 18, 2023. However, the previous rules issued in 2016 are still in effect alongside the new rules, causing confusion in ethical review work (1). Furthermore, although the Review Measures explicitly state that medical institutions at or above the secondary level, health institutions at or above the district level (including disease prevention and control centers, maternal and child health care institutions, blood collection institutions, etc.), higher education institutions, research institutes, and other institutions engaged in life science and medical research involving human subjects are required to establish Institutional Ethics Review Committees (IERC), and it outlines six basic requirements including risk control, informed consent, fairness and impartiality, free of charge and compensation, protection of privacy and personal information, and special protection (2). These six basic requirements are expanded from common truths, the four principles of autonomy, beneficence, non-maleficence and justice. This is consistent with the system used by the National Institutes of Health (NIH) in the United States or Health Research Authority (HRA) in the United Kingdom. However, it suffers from the lack of specific implementable and detailed review specification paths, and ethical review of research involving human life sciences still faces many problems.

According to a study, the IERC of a provincial hospital in China received 184 reports of protocol violations submitted by clinical trials in 1 year. Among them, 29 cases (approximately 16%) were classified as major protocol violations, including inclusion of subjects who did not meet the criteria, incorrect treatment or administration of incorrect doses, and use of combination drugs explicitly prohibited by the protocol (3). Another study on ethical review of clinical trials for anti-tumor drugs revealed that a city-level hospital in China reported a total of 2,768 protocol violations over the past 2 years (4). In contrast, Western countries have significantly lower numbers of protocol violations (5). Enhancing the quality and efficiency of ethical review in life sciences and medical research projects, ensuring transparency in the review process, and effectively preventing ethical violations in experimental plans have become urgent issues in the establishment of ethical review systems. Drawing on Michel Foucault’s concept of panopticonism, this paper incorporates the ideas of “Sign Systems” and “Discipline” into the construction of an ethical review system. It refines the ethical review mechanism using the design concept of the “Panopticon,” aiming to provide a reference for improving medical ethical review regulations in China.

## 2. Current status of China’s ethical review system

Currently, China’s ethical review system consists of “four levels of management”: the National Ethics Review Committee (NERC), the National Traditional Chinese Medicine Ethics Review Committee (NTCMERC), the Provincial Ethics Review Committee (PERC), the Regional Ethics Review Committee (RERC), and the Institutional Ethics Review Committee (IERC) (2). The “four levels of management” is a top-down approach to ethical regulation.

NERC and NTCMERC conduct research on major ethical issues in biomedical research involving human subjects, provide policy advice, and guide PERC’s ethical review work. NERC oversees and manages ethical review nationwide, while NTCMERC focuses on traditional Chinese medicine research. PERC assists in promoting the institutionalization and standardization of ethical review in provincial regions. It guides, inspects, and evaluates the work of RERCs involved in biomedical research, and provides related training and consultation. RERC is responsible for overseeing ethical review within their administrative regions and reviewing clinical trial protocols for institutions or registration applicants without internal review capabilities. IERC is responsible for conducting ethical review within their respective organizations.

However, NERC, NTCMERC, and PERC have not assumed ethical review responsibilities; they have only established departments. IERC is the main department for ethical review, and RERC may handle a limited number of research projects. Despite IERC being the primary force behind ethical review, the top-down regulatory mechanism has not been fully effective, indicating a lack of supervision and management within China’s ERCs.

For example, in an analysis of severe adverse events (SAEs) that resulted in death in subjects in a clinical trial, it was noted that the investigator changed the cause of death record for one subject from “death of unknown cause” to “death due to disease progression” without submitting a record of disease progression (6). This case reflects three serious problems: first, the investigator did not pay attention to the ethical review and arbitrarily changed the submitted materials without submitting them as evidence; second, the members of the IERC were not rigorous in their review and did not find any problems with the submitted materials, and there was a lack of tracking and guidance for the investigator; and third, there is no supervision and management from the higher-level ERC, which resulted in a lack of compliance in the review process of the IERC.

Additionally, the Review Measures stipulate that ethics committee members should include experts in the fields of biomedicine, ethics, law, and sociology, with a minimum of seven members (2). However, the lack of further details results in different professional ratios among committee members, leading to variations in the quality of implementation among ERCs. China’s ethical review system is primarily based on Western countries’ relevant systems, but due to cultural differences, it is experiencing challenges in adaptation (7). Studies on IERC by Western scholars have reported operational inefficiencies, delays in approvals affecting research project initiation, difficulties in investigator-committee member interactions, inconsistent enforcement of the same rules, and inadequate guidance for investigators (8–10).

Although the Review Measures outline the six basic requirements for biomedical research involving human subjects and specify the materials needed for ethics review, there is a lack of uniformity and clear rules across departments and regions. This means that ethical review standards and regulations are not harmonized between different levels of management, and there is a lack of mutual recognition mechanisms. Different interpretations of the Review Measures by different ERCs have resulted in divergent review practices (11). Such inconsistencies hinder top-down supervision and reduce the quality and efficiency of ethical review. During the review process, these inconsistencies manifest as incomplete documentation, inconsistent and untimely submissions, non-compliant stamps/signatures, and issues with researcher qualifications and team composition (12). For example, a scholar investigated the quality of informed consent forms in clinical research at his hospital and found that more than half (54.1%) of the 678 informed consent forms did not comply with the right regulations, including missing information about the risks of the research and the content of indemnification, as well as the use of inappropriate language (13). The main reason for this is that the IERC has not standardized the content and requirements of the informed consent form, which has resulted in a large number of poorly submitted and missing informed consent forms, further jeopardizing the subject’s right to informed consent.

In conclusion, the current flaw in the ethics review system lies in the inadequate system of ERCs (14), lack of accountability, absence of mutual monitoring mechanisms, inadequate tracking of review provisions (15), limited review transparency, and a lack of disciplinary measures within the top-down ethics regulatory system.

## 3. The connotation of panopticonism

Panopticism, originally proposed by French philosopher Michel Foucault in his book “Discipline and Punish: The Birth of the Prison,” is a sociological theory based on the architectural concept of the “Panopticon” introduced by Jeremy Bentham in the late 18th century. The so-called “Panopticon” means that there is a watchtower in the center of the open space surrounded by the outer wall of the prison building, and the watchtower has a circle of large windows facing the circular building. The circular building is divided into small cells, which can not be seen between the residents. But each cell has two windows, opposite to each other, facing the watchtower on the one hand and facing the outside on the other, so that the light in the cell is sufficient, Residents are easily identified and observed by the watchers inside the watchtower (16). Due to the bright light emitted by the watchtower, residents are unable to determine whether and when they are being monitored, resulting in an invisible discipline in the prison. Each prisoner exhibits self-discipline, fearing that someone will monitor their every move.

Foucault coins the term “Panopticism” to describe this architectural design, which serves as both a supervisory mechanism and a standard of common truth. As a supervisory mechanism, it operates through constant and cautious surveillance, meticulously recording even the slightest changes. As a standard of common truth, it categorizes and examines the surface of the body using binary logic (17). Panopticism, as the underlying principle of “political anatomy,” disciplines various relationships. The “sign” mentioned by Foucault is a technique and a means—a sign representing an “obstacle” and a “sign system” that codifies all behavior to dominate the entire field of activity. The art of discipline, according to Foucault, focuses on preventing future mistakes rather than dwelling on past sins. It utilizes a sign system to eliminate the desire to make mistakes, discouraging any potential wrongdoers (16). This constructed “Panopticon,” built through discipline and the sign system, internalizes the idea of constant surveillance and the gaze of observers, shifting from “heteronomy” to “self-discipline.” Panoptic openness, as a disciplinary mechanism of supervision and surveillance, emphasizes the constant threat of being monitored rather than actual supervision.

In the health care field, the establishment and implementation of ethical review systems aim to protect the rights and well-being of patients participating in research and clinical interventions (18), ensuring ethical standards are upheld in medical research and practice. Ethical review processes subject researchers and clinical doctors to scrutiny and observation, aligning with the ideological connotation of panopticism. However, the purpose of this monitoring and supervision is not about control and dominance but rather about ensuring adherence to ethical rules and patient welfare. The review process involves multidisciplinary teams working together to maintain transparency, accountability, and compliance with established ethical guidelines.

### 3.1. Sign systems

In his works, Foucault did not explicitly develop the concept and specific theory of “sign systems” or “sign technologies.” However, in a lecture on “Technologies of the Self” at the University of Vermont in October 1982, he mentioned “Technologies of Sign Systems” as a tool for discipline (19). In the health care system, “signs” primarily manifest in the rules and regulations of medical institutions. These explicit guidelines warn medical staff about prohibited actions or necessary measures, conveying meaning through the system. The sign system encompasses society as a whole, coding all forbidden behaviors and forming a “sign system” that serves as a spiritual cautionary line. As Foucault once stated, “There is nothing that weakens the legal system more than the fluke mentality of hoping to be lenient” (16). The sign system allows people to establish a shared truth regarding “prohibition,” a universally accepted norm that prohibits violations and must be observed. It represents an absolute authority materialized through micro-rights, creating boundaries to prevent transgressions.

The sign system operates as a consensual norm and implies continuous coercion. By tightly dividing time, space, and activity codes, the sign system not only supervises outcomes but also regulates the process of activities (16). It embodies the automation of rights (20), indicating what individuals should or should not do and the punishments that result from deviating from established routines. The significance of sign systems lies in their capacity to influence behavior and actions. Utilizing a sign system involves coding all possible scenarios, gradually transforming chaos into order, disciplining actions, institutionalizing procedures, and ultimately enhancing the efficiency and quality of practice.

### 3.2. Discipline

The disciplinary pyramid is a power structure that enables task separation, coordination, and supervision, resulting in improved efficiency. The analysis and division of time, gestures, and bodily forces constitute modes of operation (21). Discipline not only involves physical manipulation but also serves as a spiritual warning. It encompasses four techniques: the art of distributions, the control of activity, the organization of geneses, and the composition of forces.

Distribution should start from space, and the most basic operation of disciplinary rights is the analysis of space, which is usually represented as a single cell, i.e., a “tableax vivantsl” (16, 22). This classification of people and space prevents confusion, decomposes complexity, and enhances recognition and monitoring efficiency. It provides a clear measure of responsibility for individuals.

The control of activities in discipline focuses on the development rather than the outcome of activities (23). Schedules imply compulsion, i.e., the permission or prohibition of certain activities within a time unit, specifying specific matters and actions within the time unit, and the more detailed the separation of time units the easier it is to monitor, which is a tool to improve efficiency and quality. Schedules serve as symbolic signs, embodying action standardization and system concretization. For instance, this control of activity can be expressed as the nurse’s execution of medical orders within a specified time frame.

The organization of geneses refers to the integration of dispersed individual time into linear time through the serialization of continuous activities (16). It involves stringing together fragmented time through subtle decomposition and simplification. Educational activities often employ this technique, fostering ethical awareness and work systems among researchers.

The composition of forces does not pertain to physical strength but rather the precise ordering and distribution mechanisms for arranging things and individuals in personalized classifications. It aims to achieve the most efficient combinations. Rationalizing committee membership can improve the efficiency and quality of ethical review in ERCs.

Discipline also encompasses three means: hierarchical surveillance, normative ruling, and inspection.

Hierarchical surveillance relies on a structured observation system to maintain control. Higher authorities exercise supervisory authority, reinforcing discipline and preventing regulatory confusion. This hierarchical surveillance, forming a pyramid-shaped system, establishes power relations between supervisors and supervisees (16).

Normative ruling involves standardized and quantitative operations of discipline, emphasizing the distribution between positive and negative poles. Examples include the delineation of restricted areas in criminal law practice. This quantitative discipline ensures punishment aligns with the quality and quantity of violations (24). Developing a system of rewards and punishments with clear grading gaps and differentiation between “good and bad” is crucial for standardizing rulings.

Inspection combines hierarchical surveillance, normative ruling, and power rituals, force deployment, and truth establishment (16). As a disciplinary mechanism, panopticism relies on detailed inspection and supervision. Inspection involves continuous observation and supervision, creating a sense of being monitored and fostering self-discipline. It leads to internalized “gaze” (25), characterized by self-supervision and self-discipline. Inspection not only manifests as spatial surveillance, but also places people in a written network (16). Recording, registering, and classifying play decisive roles in the analysis of inspection results, providing feedback for the improvement of sign systems, truth principles, and power combinations within the discipline system.

## 4. The “panopticon” of ethical review

The ethical review “panopticon” is constructed through the interplay of the sign system and discipline mechanisms. The sign system encompasses detailed rules, while discipline entails the internal gaze and effective implementation of these rules. The sign system expands and enhances the top-down “ethical review pyramid,” building upon the macroscopic provisions outlined in the Review Measures. The discipline mechanism relies on the sign system technology and the framework of “four technologies” and “three means” of discipline to establish the “Panopticon” of ethical review.

We need to further clarify the scope of application of the panopticon system of ethical review. We need to categorize trial researches into three levels according to the risk of the researches, i.e., minimal risk, low-risk and high-risk researches. Minimal risk studies can be exempted from ethical review in order to alleviate the unnecessary burden on researchers and facilitate the conduct of life science and medical research involving human beings. Simplified ethical review can be applied to low-risk researches while high-risk researches are subject to the panopticon system of ethical review proposed by us. The above categorization refers to the specifics in Table 1 (2, 26). However, if, during the process of summary ethical review for low-risk researches, there are situations such as changes in the risk–benefit ratio of the research, disagreement among ERC members, or ERC members’ suggestion of necessitating a meeting review, the review should be adjusted to a formal one. In addition, although, our proposed panopticon system of ethical review is more applicable to the ethical review of high-risk researches, we believe that the training and education part of it is applicable to all researchers, and not only researchers involved in high-risk researches need to be trained and educated. This is a better way to maintain ethical awareness among researchers.

**Table 1:** Risk classification and scope of application of ethical review.

Risk classification of trial research	Ethical review requirements	Scope of application
Minimal risk research	Exemption from ethical review	1. Projects that utilize legally available public data, or data generated by observation and not interfering with public behavior, for research purposes
		2. Projects that use anonymized information data to conduct research
		3. Projects that use existing human biological samples for research, where the source of the biological samples used complies with relevant regulations and ethical principles, where the content and purpose of the research are within the scope of standardized informed consent, and where the activities do not involve the use of human germ cells, embryos, and reproductive cloning, chimerism, or heritable genetic manipulation
		4. Research using human cell lines or cell lines, etc. from biobank sources, where the content and purpose of the research is within the scope of the provider’s authorization and does not involve activities such as human embryos and reproductive cloning, chimerism, or heritable genetic manipulation
Low-risk research	Simplified ethical review	1. Research with no more than minimal risk
		2. Studies with minor modifications to approved research protocols that do not affect the risk–benefit ratio of the study
		3. Follow-up review of approved studies
		4. Confirmation of ethical review opinions issued by the lead institution by the ethical review committees of the participating institutions in multi-institutional studies
High-risk research	Panoramic ethical review	1. Research on the synthesis of new species that have a significant impact on human life and health, values, and the ecological environment
		2. Research related to the introduction of human stem cells into animal embryos or fetuses and their further conception into individuals in the animal uterus
		3. Basic research on altering the genetic material or patterns of inheritance in the nuclei of human germ cells, fertilized eggs and pre-implantation embryonic cells
		4. Clinical study of invasive brain-computer interfaces for the treatment of neurological and psychiatric disorders
		5. Research and development of human-computer integration systems that have a strong impact on human subjective behavior, mental emotions, and life and health
		6. Research and development of algorithmic models, applications and systems with the ability to mobilize public opinion and guide social awareness
		7. Development of automated decision-making systems with high autonomy for scenarios involving safety and health risks.

### 4.1. The sign system of ethical review

**Figure 1:**
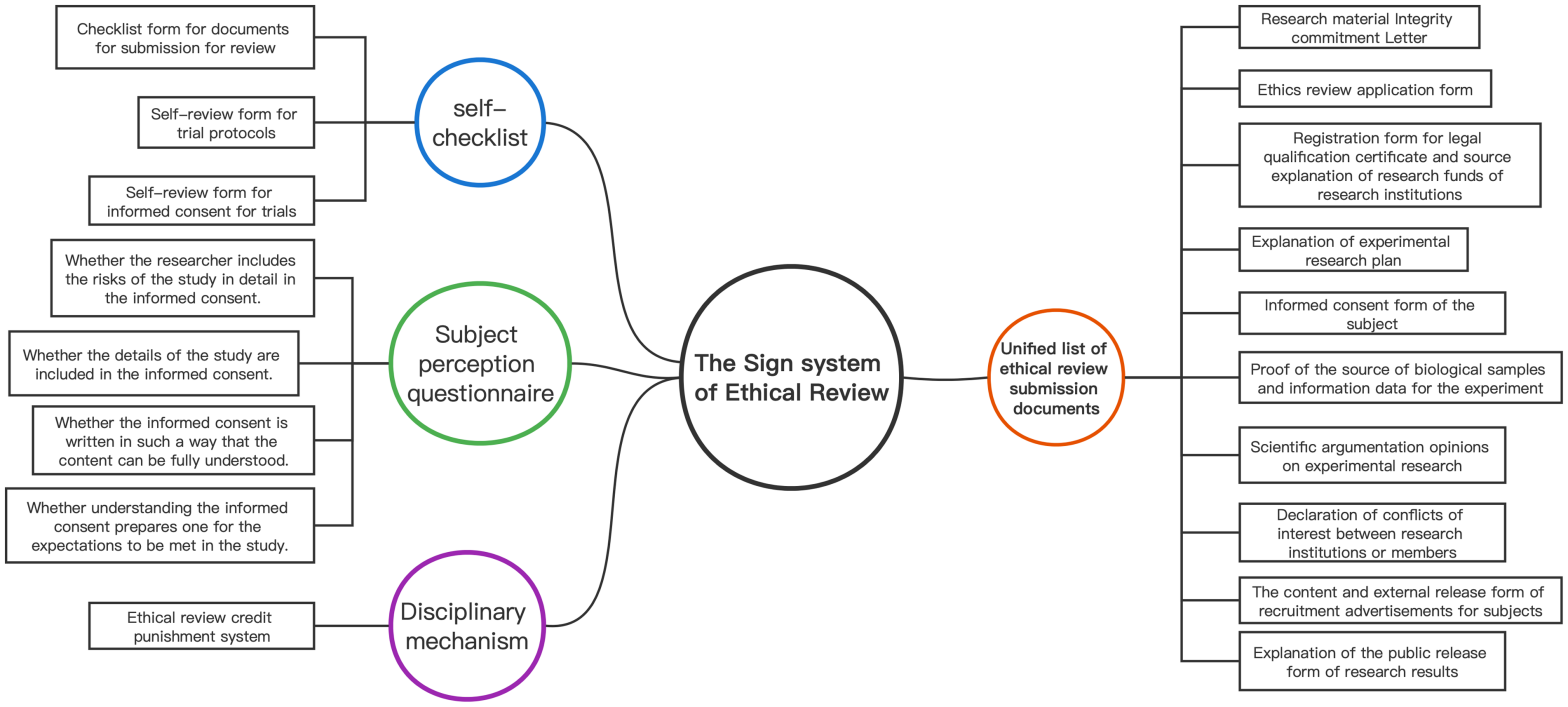
Sign system of ethical review.

The sign system plays a vital role in the “ethical review panopticon” as embodied signs that provide explicit instructions in daily life, including objects, gestures, and texts. Figure 1 presents a detailed composition of the ethical review sign system. Foucault emphasizes that signs must represent exhaustive common truths (16), which presuppose unity. While the Review Measures comprehensively list the research materials required for initial ethical review by researchers in the field of human life science and medical research, the submitted materials lack uniformity. Therefore, unifying the research materials submitted to the ERC is crucial to establish a sign system. By standardizing the document format, ethical review can approach a unified “national mutual recognition” model, allowing for nationwide recognition of audit results and optimizing the monitoring capabilities of the ethical review “panopticon.”

Second, sign as a means of heteronomy must rely on external tools to accomplish compliance with the rules. The self-checklist is a means by which an individual relies on an instrument to achieve heteronomy and exerts a mandatory normative role. Therefore, by establishing a checklist form for documents for submission for review, a self-review form for trial protocols, and a self-review form for informed consent for trials, we can achieve the purpose of standardizing the form of ethical review documents and improving the quality and efficiency of the documents submitted for review.

In addition, ethical review involves the probability of risks to the subjects, and in order to effectively monitor that informed consent signing is not a mere formality and to safeguard the subjects’ right to informed consent (27, 28), a subject perception questionnaire should also be developed and designed to cover four aspects (29): (1) whether the researcher includes the risks of the study in detail in the informed consent; (2) whether the details of the study are included in the informed consent; (3) whether the informed consent is written in such a way that the content can be fully understood; and (4) whether understanding the informed consent prepares one for the expectations to be met in the study.

To establish a sign system, the most important thing is the punishment sign, which Foucault argues enables the violation and punishment to be closely linked (16). However, due to the fact that ethical norms are often more extensive than laws, a series of ethical controversies and disagreements may arise in ethical review. Some experimental research often involves a great deal of collaboration and coordination among researchers from different disciplines and institutions, meaning that members are in a state of “moral strangers,” leading to differences in research behavior that may lead to the emergence of legitimate but unethical practices.

Furthermore, the establishment of a disciplinary mechanism is essential to address ethical misconduct and violations in the current ethical review system. While ethical review focuses on prevention and education (30), there seems to be a lack of consequences or a “cost of violation” for breaking ethical review rules. This not only weakens the deterrent effect but also allows for potential imitators who may also violate the rules. Therefore, disciplinary mechanisms must be put in place to deter unethical behavior and ensure compliance.

To establish a disciplinary mechanism, the most important thing is to follow the principle of common truth. As Foucault believes, disciplinary practice should follow the standard of common truth, or a set of obvious, justifiable and accepted norms, in addition to the principle that discipline must be based on the approximate equality of the interest levels of the normative means of adjudication (14), raising the “cost of violation.” In other words, it is necessary to quantify the violations, divide them into levels, assign points accordingly, and then determine the level of punishment according to the different violations. Establishing an ethical review credit punishment system can draw inspiration from the cumulative scoring system (total score twelve point system) of China’s motor vehicle driver’s license model. Each researcher would be allocated a certain number of points annually, and violations of ethical review rules would result in point deductions and corresponding penalties. Additionally, researchers would receive ethics education and training, and may face fines, compensation, mandatory training, or other appropriate measures. In cases of serious ethical violations, researchers should be barred from participating in any research for a specified period of time.

However, some scholars have pointed out that such means of disciplinary control, when applied to the supervision and monitoring of prisoners, can have a negative effect on the psychological development of prisoners, thereby increasing the crime rate (31). We believe that this is a psychological problem of rebellion or resistance. And this problem, when it enters into our proposed ethical review system based on panopticonism, may be the one that will lead to problems such as resentment and distrust of the ethical review system by researchers. How can a crisis of confidence between ERCs and investigators be avoided? This necessitates ensuring that both parties have the goal of protecting the interests of the subjects and ensuring that the trial is conducted safely and smoothly, which is the basis for building trust between them. We criticize those who see ERC as a game of “cat and mouse,” i.e., the members of the ethics committee are not there to help the applicant improve the list of reports submitted, but simply to make things difficult, and the applicant tries to exploit the loopholes in the review system for his or her own benefit.

We believe that the ERC has an obligation to point out any irregularities or ethical violations in the information submitted by the investigator and to assist the investigator in improving the information to ensure that the trial can be conducted safely. In addition, we have reason to believe that the investigator is not as ethical as the members of the ERC, so the investigator must adopt a positive attitude to cooperate with the ERC and revise the problems in the submitted information to avoid ethical problems and risks of the trial, and to protect the safety and interests of the trial subjects.

### 4.2. The disciplinary mechanism of ethical review

Discipline is a supervisory process, not a result, which achieves the effect of heteronomy by means of an uninterrupted and continuous sign system, and then carefully controls the functioning of the body by dividing time, space and activity codes as tightly as possible, so that the individual eventually achieves self-discipline.

The process of discipline begins with the allocation of human space, which involves structuring the members of the ERC. Given the interdisciplinary nature of ethical review, encompassing ethics, law, sociology, and biomedicine, the ERC must consist of at least 7 members, with 5 members representing diverse academic backgrounds [this value refers to the “Human Object Protection Policy” document released by the Illinois Public Health Bureau in the United States (32)]. This composition ensures a comprehensive and thorough review of research activities falling under the ERC’s jurisdiction.

To ensure a well-rounded composition and effective review, it is recommended that the committee maintain a proportion of experts in medical ethics or bioethics of more than one-third. Additionally, attention should be given to factors such as diversity, gender, and cultural background when selecting committee members. ERCs should aim for a balanced representation, avoiding exclusive male or female composition as well as exclusive representation from a single profession (32). In cases where the overall number of committee members changes, the staffing ratios mentioned above should be adjusted proportionally to maintain a reasonable composition ratio.

However, due to the fact that the members of the ERC are “moral strangers” to each other, there may be conflicts or constraints during the ethical review process. According to Foucault, it is necessary to establish hierarchical levels and utilize force composition techniques to ensure effective command and supervision within the unit (16). In the context of life science research involving human subjects, the fields of medical ethics and bioethics are of primary concern, making expert members with backgrounds in these areas the most suitable candidates for assuming the role of “veterans.” Additionally, to enhance the professional competence of the ERC, a selection threshold for member candidates should be implemented.

Once personnel space deployment is finalized using the “tableax vivants” concept, the use of schedules becomes crucial for improving top-down monitoring and inspection by the ERC. The schedule consists of two components: tracking reviews and random audits, and researchers’ reporting on their research activities. The former regulates regular supervision by the ERC, particularly the IERC, which should conduct follow-up reviews and random audits based on the progress of experimental research. Similarly, higher-level ERCs should also conduct periodic downward random audits and supervision. These follow-up and random audit measures can be seen as the watchtower of a “panopticon,” creating an internal “gaze” that invisibly supervises researchers and lower-level ethics committee members, ultimately fostering self-discipline. The latter part of the schedule includes specific timeframes for researchers to submit reports on study progress, SAEs, changes in study protocols (deviations, suspensions, terminations), and study completion.

We must also note that the ERC must also conduct periodic randomized audits (e.g., quarterly) to standardize research compliance with ethical requirements. Audits should not be limited to high-risk research, but could also be conducted by randomly selecting reports and information from low-risk research for audit. The primary purpose of auditing low-risk research is to avoid the risk that the purpose of the research will be distorted or that the risks of the research will change in a way that could jeopardize the safety of human subjects. If a researcher’s project is selected for an audit, the ERC must give 1 week’s notice of the audit to allow the researcher sufficient time to prepare and organize the information so that omissions do not interfere with the audit process.

Notably, in the event of an SAE that poses a risk to the subject’s health and life, researchers must promptly report it to the IERC. The IERC, in turn, should submit an SAE report to the RERC within 12 h [referring to Article 14 of China’s Regulations on Handling Medical Accidents (33)]. Moreover, the IERC and the research team must conduct root cause analysis seminars for SAEs, documenting the error causes and providing quarterly reports to the RERC.

Inspections often include a written feedback mechanism, which necessitates treating individuals as describable and analyzable objects, transforming them into “cases” (16). Through a personalized supervisory perspective, the characteristics, gaps, and issues of each member can be reviewed, ultimately resulting in official documents that provide feedback on the inspection outcomes. This approach, known as “case by case,” helps delineate the scope of responsibility for different research members and clarifies accountability. Personalization of regulations facilitates the implementation of disciplinary mechanisms that encourage individuals to take ownership of their actions. When ethical violations occur, this approach enables ERC members to identify the responsible parties and take appropriate measures to address misconduct, fostering a culture of accountability within the research community. In addition, the results of the inspection can complement the possible fallacies of the ethical review system and disciplinary mechanism by feeding back into the sign system.

Finally, in order to achieve true discipline, the internalization of ethical review systems and regulations must be accomplished through education. The organization of genes, as an educational means, simplifies complex content through decomposition, and then concatenates the simplified content through combination to achieve the inner gaze of rules. The ERC has the responsibility of educating researchers on ethical awareness, the provisions of ethical principles and the normative details of ethical review. Emanuel et al. have pointed out that clinical researchers need to be skilled in appropriate methods of clinical trials, statistical testing, outcome measurement and other scientific aspects, in addition to ethics-related training, such as subject selection standards, assessment of risk–benefit ratios, presentation of information in an appropriate manner, and confidentiality of information and ethical sensitivity (34). This proposal could be adapted to the educational aspect of disciplinary mechanisms. Therefore, given that ERC members come from different academic fields of requirements, and the contents of ethical principles and regulations and ethical review norms also involve different aspects such as medicine, ethics, sociology, law, etc., researchers are trained in stages by decomposing the content of education, and then they are finally examined by means of aggregation. The training can be conducted by online education, while the examination is conducted on an annual basis, and the results of the examination should also be linked to the score of the ethical review credit punishment system, ultimately achieving the purpose of strengthening ethics education and internalizing ethical rules.

## 5. Conclusion

China has been and will be in an era of high development of biomedical technology for a long time, but the construction of ERCs has not been able to keep pace with the development of biomedical technology, and the quality and efficiency of ethics review still cannot meet the needs of scientific research. Foucault’s concept of Panopticism provides a new perspective and path for improving the construction of ethical review systems. The framework of the Panopticon for ethical review can not only restrain the behaviors violating the ethical provisions in scientific research, but also form the internalized “gaze” and deterrence of rules.

Although this paper has proposed the construction of the sign system and discipline mechanism of ethical review, the content of the Panopticon of ethical review is still huge and extensive, and more detailed discussions still need to be further expanded. Therefore, there is a need to further fill the loopholes in the sign system and improve the suitability of the regulatory mechanism to the reality of the review, with a view to structuring the Panopticon of ethical review in accordance with the national conditions of scientific research in China.

## Author contributions

LW: Conceptualization, Methodology, Writing – original draft. XK: Supervision, Writing – review & editing.
